# OPIOID PRESCRIBING AND OPIOID USE IN OLDER ADULTS: BUILDING AND REFINING A CHANGE PACKAGE FOR PRIMARY CARE

**DOI:** 10.1093/geroni/igad104.2924

**Published:** 2023-12-21

**Authors:** Ellen Childs, Megan Noel, Clarissa Hsu, Michael Parchman, Kendall Hall, Sarah Shoemaker-Hunt, Nicole Keane

**Affiliations:** Abt Associates, Rockville, Maryland, United States; Abt Associates, Rockville, Maryland, United States; Kaiser Permanent Washington Health Research Institute, Seattle, Washington, United States; Kaiser Permanent Washington Health Research Institute, Seattle, Washington, United States; Abt Associates, Rockville, Maryland, United States; Abt Associates, Cambridge, Massachusetts, United States; Abt Associates, Cambridge, Massachusetts, United States

## Abstract

Opioid use is associated with increased risk of harms in older adults (≥65) due to physiologic changes, drug interactions, and comorbidities. The AHRQ Opioids in Older Adults project developed a change package based on an evidence review and expert recommendations that included nine high-leverage changes (HLCs) and related key activities to address safe opioid use in this population. We used the change package to support fourteen primary care practices participating in two fifteen-month learning collaboratives convened to implement innovations in safer opioid use in older adults. Given the nascent understanding of how practices address opioid safety among older adults, this presentation will describe the HLCs implemented by practices compared to the original change package offered to them as a way to validate and improve the change package for broad use and uptake. Of the original nine HLCs, practices implemented seven, including staff education, risk assessment, opioid use disorder assessment and treatment, processes and workflows, non-pharmacologic pain management, medication management, and using data to inform practice. All activities fit into an original HLC, however the activities that practices implemented varied from the original key activities identified by the literature review and expert panel. For example, under the using data to inform practice HLC, most practices struggled to complete chart reviews, and were unable to “review data regularly” as offered in the original change package. This presentation will describe strategies used to improve opioid safety for older adults in primary care.

